# Investigating Older Adults' Use of a Socially Assistive Robot via Time Series Clustering and User Profiling: Descriptive Analysis Study

**DOI:** 10.2196/41093

**Published:** 2024-09-19

**Authors:** In-jin Yoo, Do-Hyung Park, Othelia EunKyoung Lee, Albert Park

**Affiliations:** 1 Graduate School of Business IT Kookmin University Seoul Republic of Korea; 2 School of Social Work University of North Carolina Charlotte Charlotte, NC United States; 3 Department of Software and Information Systems University of North Carolina at Charlotte Charlotte, NC United States

**Keywords:** socially assistive robot, older adults, robot use pattern, time series clustering, profiling analysis

## Abstract

**Background:**

The aging population and the shortage of geriatric care workers are major global concerns. Socially assistive robots (SARs) have the potential to address these issues, but developing SARs for various types of users is still in its infancy.

**Objective:**

This study aims to examine the characteristics and use patterns of SARs.

**Methods:**

This study analyzed log data from 64 older adults who used a SAR called Hyodol for 60 days to understand use patterns and their relationship with user characteristics. Data on user interactions, robot-assisted content use, demographics, physical and mental health, and lifestyle were collected. Time series clustering was used to group users based on use patterns, followed by profiling analysis to relate these patterns to user characteristics.

**Results:**

Overall, 4 time series clusters were created based on use patterns: helpers, friends, short-term users, and long-term users. Time series and profiling analyses revealed distinct patterns for each group. We found that older adults use SARs differently based on factors beyond demographics and health. This study demonstrates a data-driven approach to understanding user needs, and the findings can help tailor SAR interventions for specific user groups.

**Conclusions:**

This study extends our understanding of the factors associated with the long-term use of SARs for geriatric care and makes methodological contributions.

## Introduction

### Overview

The costs of geriatric care and the shortage of home care workers are major global concerns [1]. Socially assistive robots (SARs) have the potential to address these issues, but developing SARs for various types of users is still in its infancy. A majority of previous research has focused on physical human-robot interaction, with an emphasis on the embodiment, control, and safety checks of robots [2]. Recent research has delved into social human-robot interaction, focusing on the robot’s communication, emotional exchange, and relationship formation [3-5].

SARs can perform the functions of emotional exchange, medical assistance, cognitive training, and activity detection. Various research efforts are now exploring SARs to resolve personal problems related to the deterioration of physical and mental health and social exclusion, especially targeting socially vulnerable groups, including socially isolated older adults. Multiple studies have confirmed the positive effects of SARs on older users including fall prevention, relief of stress and dementia symptoms, and improved quality of life [6-9].

Studies are still limited to the observation of cross-sectional user characteristics over a short period of time or to the superficial comparison of the effects before and after the introduction of SARs [10,11]. Few studies are dedicated to analyzing behavioral changes and psychological mechanisms of potential users of SARs (see [12] for an exception). This is mainly due to the high costs involved in observing user behaviors and the difficulty of confining the scope of target behaviors. Given that elder care using SARs could be a long-term solution [13], further studies are needed to analyze user characteristics and to explore the changes in interactive behavioral patterns of the users toward SARs through time series data.

### Background

#### Hyodol SAR

**Figure 1:**
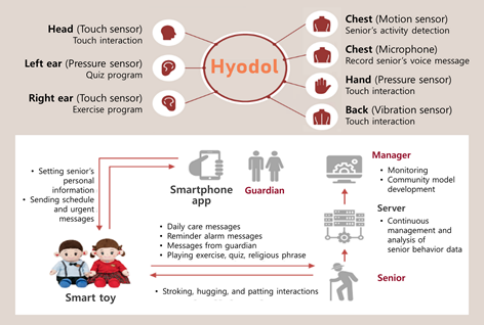
Hyodol and its features.

The data used in this study were collected and processed by Hyodol SARs (Figure 1), a companion robot for home care launched by Hyodol Ltd. This 20-inch doll, designed to simulate a grandchild, incorporates artificial intelligence and body sensors for communication [14,15]. Hyodol can motivate users to exercise and create positive social engagement. Two of its most used functions by users are (1) the interactive relationship and (2) robot-assisted contents.

The interactive relationship between Hyodol and the user is enabled by the robot’s verbal reactions to the user’s actions such as petting and holding its hand. Hyodol’s small, soft body allows people to hold, snuggle, and exchange conversation with it, enabling multimodal communication. This SAR can be customized and programmed to remind users of meals, medication, and appointments, and encourages social interaction. For example, Hyodol asks users to hold their hands after they take their medication. By touching its ears and hands, users can choose to interact with the robot in a variety of ways including listening to popular songs, playing quiz games, and following easy-to-follow exercises. Robot-assisted contents are designed to improve the user’s daily living and to exercise cognitive ability. This feature allows Hyodol to communicate health information, cue reminiscences, quote inspirational passages, and tell stories.

#### Time Series Clustering and Profiling Analysis

Cluster analysis is an unsupervised learning method that groups objects into clusters based on their similarity; it is an exploratory analysis that identifies clusters within the sample based on a high level of homogeneity and similarity [16-18].

Identifying the similarity of time series data is a method used in many different fields. Some examples include identifying users with similar use histories [19]. The similarity or dissimilarity between data points is quantified using “distance,” which can be obtained using a wide range of distance scales depending on the nature of data and analytical goals. However, when it comes to time series data, the concept of distance based on similarity cannot be easily applied due to the temporal dynamic characteristics. Similarity measures used in existing cluster analyses have limitations. Mainly, the similarity measure does not consider the interdependent relationship between time series data. To overcome this limitation, a number of researchers have tried various approaches to cluster by defining similarities and differences between time series datasets. Two such approaches are the raw data approach and the feature-based approach [20]. The feature-based approach proves effective when digital signals with specific cycles are given as a dataset, while the raw data approach is useful for calculating the similarity that reflects dynamic characteristics from time series datasets [21].

Profiling analysis is a technique typically used to quantify individual characteristics. It can be helpful to understand different types of SAR users by generating descriptions and explanations of data in this study [22]. Profiling the use pattern can reveal new attributes underlying pattern differences that the deductive approach has not been able to detect.

### Research Questions

Previous studies have not addressed SAR use patterns and behaviors for socially isolated older adults. This study aims to explore the use patterns of SARs based on time series use log data of 2 main features: interactive relationship and robot-assisted contents. The study will then cluster users with similar use patterns according to the time spent with SARs and describe characteristics from each cluster through profiling of these user groups.

## Methods

### Data Collection and Processing

The log data, collected from November 30, 2017, to February 1, 2019, consists of the frequency of users’ interaction with the robot and their use of robot-assisted contents. The data were collected at 5-minute intervals for each participant for 60 days. However, the data collection period exceeds 60 days because participants did not start using SAR at the same time. We focus on the first 60 days of use for all users. Logs that were deemed as errors were excluded from the data, resulting in 870,000 valid logs for analysis.

We first compiled time series datasets by reconstructing the log data at an individual level and processing the use data according to function and related use rates. To understand use patterns, we analyzed daily interaction time by aggregating the 5-minute data to represent the total interaction time for each day. We then used time series clustering to categorize users into different groups based on their SAR use patterns. For each cluster, we optimized time series models to confirm the characteristics of use patterns at the individual level. In the second phase, we conducted a profiling analysis to understand the differences in the use pattern in relation to individuals’ demographic, emotional, and behavioral traits.

### Participants

The recruitment process was crucial for this study given that Hyodol is designed specifically for the socially isolated population of older people, not for the general public. Our inclusion and exclusion criteria were carefully formulated to ensure that participants were well-suited for interaction with Hyodol and could meaningfully contribute to our understanding of SAR use among older adults. A video description of Hyodol can be found in Multimedia Appendix 1.

This study focused on a specific group of older adults for participation. Inclusion criteria required participants to be 70 years of age or older with no noticeable vision or hearing impairments. This ensured they could fully interact with the content and features offered by Hyodol. Additionally, sufficient mobility was necessary to use functions like exercise programs. To avoid potential disruptions or negative reactions, those with pets were excluded from the study. Finally, participants were recruited from community welfare centers to benefit from the oversight and support provided by social welfare organizations.

These recruitment restrictions, while potentially limiting the generalizability of our findings, allowed us to minimize the impact of external variables such as disabilities among participants. It is important to note that these criteria were established not only to ensure the safety and well-being of the participants but also to enhance the reliability and validity of our findings by selecting individuals who could fully engage with the SAR’s capabilities. We have clarified this in the Participants section within the Results section.

We surveyed and collected use data from 80 older adults who used SAR. We excluded users who experienced the SAR’s sensor errors and therefore lacked use log data, as well as participants who did not complete surveys. The final dataset consisted of 64 older adults who responded to both pretest and posttest surveys with enough use data. This study focuses on the longitudinal datasets collected through the functions of “Interactive Relationship” and “Robot-assisted Contents.” We selected these two functions since they can effectively reflect the user’s behaviors that are related to their physical and psychological satisfaction. Informed consent was obtained for each participant for the survey data.

The appropriate sample size was estimated using GPower 3.1 [23] software for an independent sample 2-tailed *t* test. Assuming a significance level of α=.05, an expected effect size of 0.88, and statistical power of 0.79, the minimum sample size was estimated to be 64 for clusters A and B. Similarly, for an independent sample 2-tailed *t* test using GPower 3.1 software, with a significance level of α=.05, an expected effect size of 0.88, and statistical power of 0.8, the minimum sample size was estimated to be 60 for clusters C and D. Therefore, a sample size of 64 appears to be adequate.

### Survey Measures

#### Overview

To profile the respondents, demographic survey questions were asked including gender, age, and socioeconomic status. Respondents were also asked if they received public welfare assistance from the local government, and whether they used emergency monitoring services.

#### Physical Health

Participants rated their health on a 3-point scale (0=poor, 0.5=same as before, and 1=great). Using the 3-point scale, they rated the frequency of spending time alone (0=little, 0.5=somewhat, 1=a lot). We also asked participants to report the number of chronic diseases and the number of daily medications.

#### Lifestyle Management

On a 3-point scale (0, 0.5, and 1), each participant was asked about their daily activities. These included the completion of eight daily activities such as (1) wake-up, (2) daily ventilation, (3) medication intake, (4) eating meals, (5) taking a walk outside, (6) exercise or fitness, (7) thinking positively, and (8) initiating social connection.

#### Mental Health—Depression

The Geriatric Depression Scale was used [24]. Each Geriatric Depression Scale item was rated as either yes=1 or no=0 by the respondents; the total scores (ranging from 0 to 15) were calculated. A score of 5 or higher is regarded as symptomatic of depression.

#### Performance Evaluation

Participants were asked to evaluate Hyodol’s performance with its functionality on a 5-point Likert scale. The 11 functions for lifestyle management were listed including wake-up, bedtime, daily inspirational passage, weather, meal time, time for medication, exercise or fitness, brain training games, songs, responsiveness, safety monitoring, and voice message from caregivers. The average score was used for our analysis.

#### Satisfaction

Participants were asked to rate their satisfaction with Hyodol on a 5-point scale. The following 5 aspects were assessed including usability, preference for the SAR appearance, accuracy of voice, content, and touch. The questionnaires used in this study can be found in Multimedia Appendix 2.

### Data Analysis

This study used log data from 2 main features—interactive relationships and robot-assisted contents—to examine the characteristics and use patterns of SARs. The raw data approach of time series clustering was used to investigate individual differences in use patterns. Specifically, the techniques of dynamic time warping and global alignment kernel (GAK) were considered and explored. These 2 techniques, which are algorithms for measuring pattern similarity between two temporal sequences, compare two time series regardless of the time of observation by calculating the fit between them [25]. This method enables measurements or calculations even when there are different time points of observation or different sample sizes in the two series.

After experimenting with GAK and dynamic time warping, we chose GAK for similarity scaling [25]. We specifically used the “dtwclust” package provided by the R software [26] to conduct time series clustering. The package is optimally designed for time series data clustering [26]. For the package-based time series clustering, we used GAK for the similarity scale and partitioning around medoids for the center extraction as the parameters for clustering. Following the time series clustering, we described user types based on use patterns, lifestyle, and related health behaviors via profiling analysis. Use patterns include interaction (eg, stroking, handholding, and petting) and content use (eg, exercise or fitness and brain training).

### Ethical Considerations

This project has been reviewed by the Kookmin University Institutional Review Board (KMUIRB; KMU-202304-HR-348) for the secondary analysis of users’ log data. Informed consent was read to each participant, and signatures were obtained from each research participant. Participant identities were protected through the complete anonymization of all data used in this research. To reduce bias, participants were not compensated financially for their participation.

## Results

### Time Series Clustering

Table 1 summarizes the amount of interaction and content use for each participant. The participants were divided into 2 clusters for each type of use (interaction and content) based on the amount of use. We chose to compare 2 specific clusters with sufficient data for robust analysis. Based on the use patterns for interaction, users were categorized into two clusters of 51 and 13 individuals. Users were classified into two clusters of 49 and 15 individuals based on the use patterns for contents. It is important to note that only clusters A and B within interaction use and clusters C and D within content use will be compared with each other. This is because the study was designed to compare use patterns within each type of use (interaction and content), rather than to compare use patterns across types of use.

**Table 1:** Time series clustering results.

	Interaction use		Contents use	
	A	B	C	D
Intracluster distance	0.010	0.017	0.000	0.000
Size, n (%)	51 (80)	13 (20)	49 (77)	15 (23)

In Table 2, we summarize overlapping membership to explore connections between activities within clusters. Table 3 shows the general participant information regarding their age, gender, health, and social tendencies.

**Table 2:** Associations between activities within clusters.

Interaction use	Function use		Total, n (%)
	C	D	
			
A	45	6	51 (80)
B	4	9	13 (20)
Total, n (%)	49 (77)	15 (23)	64 (100)

**Table 3:** General participant characteristics for each cluster.

			Cluster name									Total
			AC		AD		BC		BD			
Participants, n			45		6		4		9		64	
Sex (female), mean (SD)			0.60 (0.40)		1.00 (0.000)		1.00 (0.000)		0.89 (0.33)		0.70 (0.46)	
Average age (years), mean (SD)			78.58 (8.07)		77.17 (10.52)		85.50 (3.70)		82.33 (6.63)		79.41 (8.07)	
Subjective health, mean (SD)			0.47 (0.42)		0.417 (0.38)		0.25 (0.50)		0.44 (0.46)		0.45 (0.42)	
Alone time, mean (SD)			0.43 (0.35)		0.583 (0.20)		0.75 (0.50)		0.72 (0.36)		0.51 (0.36)	
Number of diseases, mean (SD)			2.16 (1.51)		2.17 (0.98)		2.00 (0.82)		2.00 (1.00)		2.13 (1.35)	
Number of medicines, mean (SD)			2.24 (1.32)		2.17 (0.75)		2.75 (0.50)		2.00 (0.87)		2.23 (1.18)	
**Lifestyle management, mean (SD)**												
	Daily ventilation	0.64 (0.38)		0.75 (0.27)		0.63 (0.48)		0.44 (0.39)		0.63 (0.38)		
	Regular meals	0.59 (0.42)		0.67 (0.41)		0.38 (0.25)		0.28 (0.36)		0.54 (0.41)		
	On-time medicine intake	0.54 (0.40)		0.75 (0.27)		0.50 (0.000)		0.39 (0.42)		0.54 (0.38)		
	Wake-up	0.53 (0.42)		0.42 (0.49)		0.50 (0.0000)		0.39 (0.42)		0.50 (0.41)		
	Walks outside	0.47 (0.40)		0.67 (0.26)		0.50 (0.0000)		0.33 (0.43)		0.47 (0.39)		
	Exercise or fitness	0.42 (0.41)		0.42 (0.20)		0.63 (0.48)		0.22 (0.36)		0.41 (0.40)		
	Positive thinking	0.58 (0.43)		0.50 (0.32)		0.50 (0.41)		0.39 (0.42)		0.54 (0.41)		
	Social connection	0.61 (0.40)		0.75 (0.42)		0.75 (0.29)		0.28 (0.44)		0.59 (0.41)		
	Depression	5.90 (4.07)		5.00 (3.03)		8.25 (4.92)		6.11 (5.65)		6.00 (4.24)		

Cluster AC, the largest group, consisted of 45 participants. The average age of the participants in this cluster was 78.58 (SD 8.07), and it had the lowest proportion of women (n=27, 60%). On average, these participants reported 2.16 (SD 1.51) chronic diseases and took 2.24 (SD 1.32) medications. They rarely spent time alone, with an average of 0.433 (SD 0.35) hours per day which indicates a moderate level of depression scores (mean 5.90, SD 4.07).

Cluster BC comprised 4 women, making it the smallest group. This group is also the oldest, with an average age of 85.5 (SD 3.70). These users spend the largest amount of time alone (mean 0.750, SD 0.50). On average, they had two chronic diseases and took 2.75 (SD 0.50) medications. Notably, they had the highest depression score (mean 8.25, SD 4.92) among all the clusters.

Cluster BD consisted of 9 users, with 89% (n=8) being women. The average age of this cluster was 82.33 (SD 6.63), making them older than AC and AD, but younger than BD. They reported a relatively low number of chronic diseases (mean 2.00, SD 1.00) and daily medications (mean 2.00, SD 0.87) and spent many hours alone on a daily basis (mean 0.72, SD 0.36). Furthermore, they exhibited a moderate level of depression scores (mean 6.11, SD 5.65).

**Figure 2:**
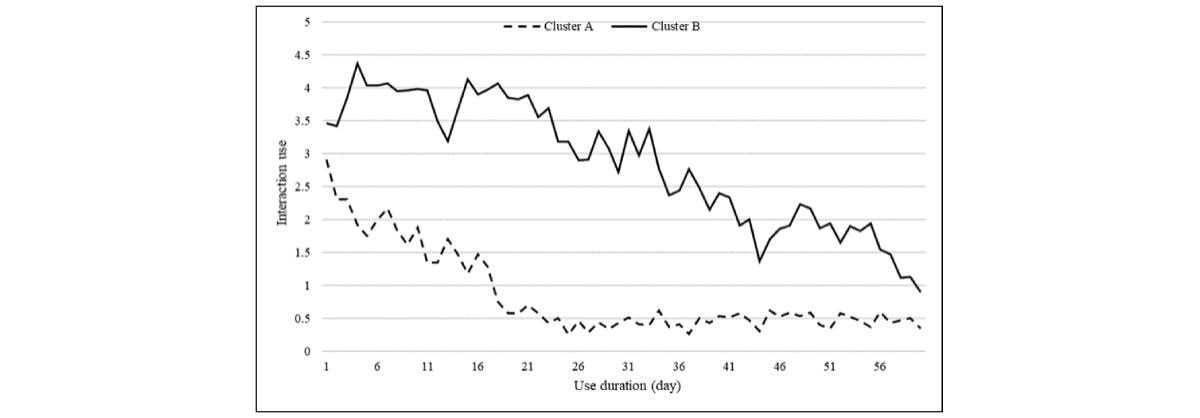
Cluster A, B: interaction use centroid.

Although the clustering results provide a general picture of each cluster’s characteristics, specific differences in use traits are not known. Thus, the centroids of use for each cluster were calculated to identify clear patterns. The centroids of use and corresponding graphs are shown in Figure 2.

Cluster analysis for interaction use shows that users grouped in cluster A have a lower use level than users in cluster B. Their interaction use sharply declines within the first 21 days of use, while the users in cluster B show a gradual decrease in use for the entire period of 60 days. The average use of cluster B users is also higher than that of cluster A users.

Content-based use clusters C and D show that users in cluster C had less average use time than users in cluster D. However, both clusters display a very low average use after 41 days. Users in cluster D used more content than users in cluster C. Content use for cluster C users dramatically decreased after the initial 5 days of use and remained at a very low level. On the other hand, content use for cluster D users gradually decreased for 45 days but remained higher than that of cluster C users.

### Time Series Analysis

The use time steadily decreases for all 4 groups. With the assumption that users will eventually stop using SAR, we used time series analysis to identify patterns and examine their traits computationally. The results from applying a damped-trend model [27-29] to the four clusters are presented in Table 4.

**Table 4:** Applying damped-trend and Holt model result.

Model			Estimate, mean (SE)	*t* test *(df)*	*P* value
**Interaction**					
	**Cluster A**				
		α	0.418 (0.103)	4.071 (57)	<.001
		γ	0.129 (0.102)	1.267 (57)	.21
		π	0.966 (0.033)	29.457 (57)	<.001
	**Cluster B**				
		α	0.700 (.124)	5.626 (58)	<.001
		γ	0.000 (.020)	0.003 (58)	.99
**Content**					
	**Cluster C**				
		α	0.500 (0.113)	4.423 (57)	<.001
		γ	0.513 (0.390)	1.316 (57)	.19
		π	0.835 (0.117)	7.113 (57)	<.001
	**Cluster D**				
		α	0.067 (0.035)	1.925 (58)	.06
		γ	0.415 (0.223)	1.861 (58)	.07

The damped-trend model was applied to clusters A and C. The models for clusters B and D evolved from the primary model to the Holt model consisting of α and γ. For cluster B, the significance probability of the trend factor of γ is *P*=.99, meaning that the time series use data in the cluster follows simple linear exponential smoothing. Finally, optimal models for each group’s time series datasets were constructed: the damped-trend model for cluster A, simple linear exponential smoothing for cluster B, the damped-trend model for cluster C, and the Holt model for cluster D. Each group’s specific characteristics are described in the following sections.

Cluster A has no seasonality, and its linear trend gradually disappears; the temporal mean of the time series data follows the trend and ultimately approximates a constant. Based on parameter estimates, cluster A’s SAR use showed no trend changes (γ, *P*=.21), and its amplitude declined over time. Cluster A’s smoothing factor of α, estimated by the damped-trend, is 0.418 with a *t* value of 4.423 and *P*<.001. This result indicates that more weight is placed on the values observed earlier than the most recently observed values. The estimated value of π, the factor of decreasing amplitude trend, is 0.966 with a *t* value of 29.457 and *P*<.001. When considering π’s maximum value of 0.98 in real-world situations, the effect on amplitude reduction was minimal, which translates into a gradually declining amplitude of use, and ultimately, no use in the end.

Cluster B has no trend and seasonality in which temporal changes in the average amount within a time series follow a horizontal pattern. Therefore, as shown in Figure 2, it is assumed that cluster B’s SAR use amount takes the form of a horizontal line with few temporal changes. Cluster B’s smoothing factor of α, estimated by the simple exponential smoothing, is 0.700 with a *t* value of 5.988 and *P*<.001. This result indicates that more weight is placed on the values observed earlier than the most recently observed values. Cluster B’s use graph may create an illusion of a downward trend. However, with the selection of simple exponential smoothing as an optimal model, no actual trend cycle and seasonality were observed, and the average use was shown to be linear. This indicates that while cluster B’s use seems to follow a downward trend, it actually exhibits a linear horizontal line without a trend, which implies that SARs are steadily used for a relatively longer time.

**Figure 3:**
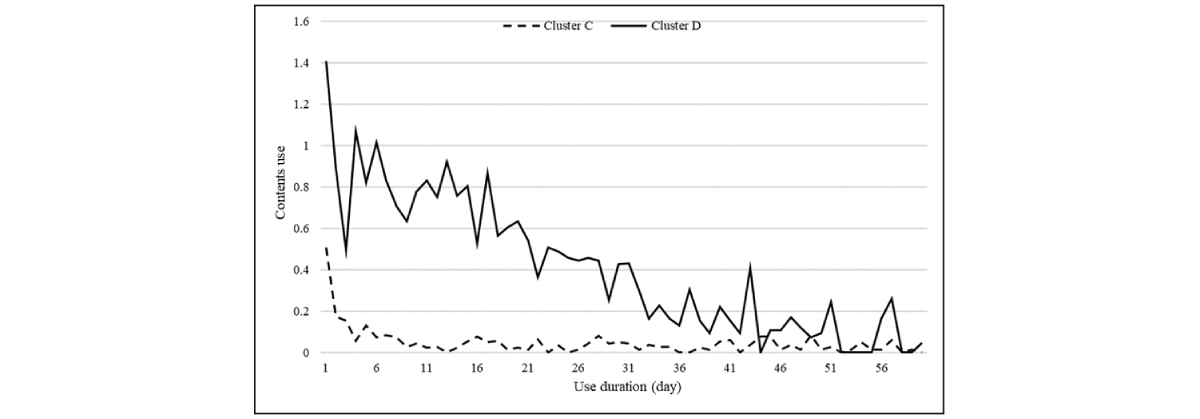
Cluster C, D: contents use centroid.

Cluster C’s SAR use displays no trend changes (γ, *P*=.19), and the amplitude decreases over time. The cluster’s use shows an exponential function and gradually approaches zero in relation to the damped-trend, as shown in Figure 3. Cluster C’s smoothing factor of α, estimated by the damped-trend, is 0.500, with a *t* value of 4.423 and *P*<.001. This result indicates that equal weight is placed on both the values observed earlier and the most recently observed values. The estimated value of π, the factor of the decreasing amplitude trend, is 0.835, with a *t* value of 7.113 and *P*<.001. Since π’s value is rarely below 0.8 in real-world situations, the effect on amplitude reduction is significant. In other words, the use-related amplitude reduction is immediately observed, with the use approximating a constant thereafter. This indicates that the use sharply declines to almost no use over a short period.

Cluster D’s use changes over time (Figure 3). Cluster D’s estimated smoothing factor of α is 0.067, with a *t* value of 1.925 and *P*=.06. This result indicates that more weight is placed on the values observed earlier than the most recently observed values. Cluster D’s estimated trend factor of γ is 0.415 with a *t* value of 1.861 and *P*=.07, which indicates that Cluster D’s use follows the Holt model with the acceptance level of α and γ at *P*=.10. Due to the model’s nature, it is observed that the overall trend is present with no cycle or seasonality. The Holt model for cluster D confirms the decreasing trend, which appears on the graph of SAR use, actually exists.

### Profiling Analysis

#### Interaction Use

The demographic characteristics of clusters A and B users are summarized in Table 5. To address multiple comparisons in profiling analysis, we applied *P* value adjustments using the Hommel procedure [29] in Tables 5-8. Cluster A consists mainly of men (n=18, 35%) and women (n=33, 65%) aged younger than 80 years old. Those who receive public welfare assistance from the local government accounted for 43% (n=22). Their perceived health status is somewhat negative, with 37% (n=19) of them rating their health as “poor.” They also spend less time alone (n=14, 27%). Cluster A users lead relatively balanced lives as evidenced by all the items of the life management survey. These users ventilate their rooms daily, get up and eat on a regular basis, take their medicine, walk and exercise more frequently, have more positive thoughts, and have a low dependence on SARs. Since they enjoy good mental health and have less emotional interaction with the robots, for the rest of the paper, we will refer to this group as “SARs as Helpers.”

**Table 5:** Descriptive statistics of interaction use cluster.

Variables		Cluster A (n=51)	Cluster B (n=13)	*t* test *(df)*	*P* value	Adjusted *P* value
Sex (female), n (%)		33 (65)	12 (92)	3.893 (62)	.05	.72
Public assistance, n (%)		22 (43)	5 (38)	0.300 (62)	.77	.99
Average age, mean (SD)		78.41 (8.283)	83.31 (5.921)	3.998 (62)	.05	.70
Subjective health, mean (SD)		0.46 (0.41)	0.39 (0.46)	0.339 (62)	.56	.99
Alone time, mean (SD)		0.45 (0.33)	0.73 (0.39)	6.762 (62)	.01	.19
Number of diseases, mean (SD)		2.16 (1.4)	2 (0.9)	0.371 (62)	.71	.99
Number of medicines, mean (SD)		2.24 (1.2)	2.23 (0.8)	0.016 (62)	.99	.99
**Lifestyle management, mean (SD)**						
	Daily ventilation	0.52 (0.42)	0.42 (0.34)	1.343 (62)	.18	.92
	Regular meals	0.66 (0.37)	0.50 (0.41)	2.353 (62)	.02	.32
	On-time medicine intake	0.57 (0.39)	0.42 (0.34)	1.234 (62)	.22	.96
	Wake-up	0.60 (0.41)	0.31 (0.33)	0.758 (62)	.45	.99
	Walks outside	0.49 (0.39)	0.39 (0.36)	0.876 (62)	.38	.99
	Exercise or fitness	0.42 (0.39)	0.35 (0.43)	0.608 (62)	.55	.99
	Positive thinking	0.57 (0.41)	0.42 (0.40)	1.142 (62)	.26	.99
	Social connection	0.63 (0.40)	0.42 (0.45)	1.610 (62)	.11	.87
	Depression	5.79 (3.93)	6.77 (5.32)	–0.734 (62)	.47	.98

**Table 6:** Subfunction use ratio by interaction use cluster.

Variables		Cluster A (n=51)	Cluster B (n=13)	*t* test (*df*)	*P* value	Adjusted *P* value
Use day, mean (SD)		31.67 (24.98)	50.00 (12.75)	6.512 (62)	.01	.04
Use day or retention period, mean (SD)		0.52 (0.27)	0.74 (0.16)	8.270 (62)	.006	.03
**Interaction, mean (SD)**						
	Stroking per day	32.5 (109.14)	26.23 (19.46)	0.042 (62)	.84	.84
	Handholding per day	6.08 (12.58)	26.58 (38.2)	10.608 (62)	.002	.02
	Petting per day	5.96 (9.14)	25.08 (48.37)	7.281 (62)	.009	.04
**Content** **, mean (SD)**						
	Exercise per day	0.25 (0.41)	0.88 (1.31)	8.605 (62)	.005	.03
	Quiz game per day	0.52 (0.68)	1.39 (2.01)	6.761 (62)	.01	.04
	Hyodol performance	4.37 (0.56)	4.78 (0.15)	5.798 (62)	.02	.05
	Hyodol satisfaction	2.66 (0.36)	2.9 (0.14)	4.701 (62)	.04	.07

**Table 7:** Descriptive statistics of contents use cluster.

Variables			Cluster C (n=49)		Cluster D (n=15)		*t* test (*df*)		*P* value	Adjusted *P* value
Sex (female), n (%)			31 (63)		14 (93)		5.225 (62)		.03	.43
Public assistance, n (%)			20 (5)		7 (47)		0.396 (62)		.69	.98
Average age, mean (SD)			79.14 (8.016)		8.27 (8.455)		0.220 (62)		.64	.98
Subjective health, mean (SD)			0.45 (0.42)		0.43 (0.42)		0.016 (62)		.90	.98
Alone time, mean (SD)			0.46 (0.37)		0.67 (0.31)		3.95 (62)		.05	.77
Number of diseases, mean (SD)			2.14 (1.45)		2.07 (0.96)		0.190 (62)		.85	.98
Number of medicines, mean (SD)			2.29 (1.27)		2.07 (0.80)		0.796 (62)		.43	.98
**Lifestyle management, mean (SD)**										
	Daily ventilation	0.53 (0.40)		0.40 (0.43)		0.680 (62)		.50		.98
	Regular meals	0.64 (0.38)		0.57 (0.72)		1.141 (62)		.26		.98
	On-time medicine intake	0.54 (0.38)		0.53 (0.40)		0.066 (62)		.95		.98
	Wake-up	0.57 (0.41)		0.43 (0.42)		1.086 (62)		.28		.98
	Walks outside	0.47 (0.39)		0.47 (0.40)		0.024 (62)		.98		.98
	Exercise or fitness	0.44 (0.42)		0.30 (0.32)		1.188 (62)		.24		.98
	Positive thinking	0.57 (0.42)		0.43 (0.37)		1.141 (62)		.26		.98
	Social connection	0.62 (0.39)		0.47 (0.48)		1.283 (62)		.20		.98
	Depression	6.11 (4.14)		5.67 (4.67)		0.348 (62)		.73		.98

**Table 8:** Subfunction use ratio by contents use cluster.

Variables		Cluster C (n=49)	Cluster D (n=15)	*t* test (*df*)	*P* value	Adjusted *P* value
Use day		34 (25.83)	39.93 (17.34)	0.692 (62)	.41	.67
Use day or retention period		0.51 (0.27)	0.73 (0.17)	8.911 (62)	.004	.02
**Interaction**						
	Stroking per day	34.11 (111.15)	21.8 (2.25)	0.180 (62)	.67	.67
	Handholding per day	4.76 (5.07)	28.15 (39.88)	16.583 (62)	<.001	<.001
	Petting per day	4.98 (6.73)	25.71 (45.46)	9.832 (62)	.003	.02
**Content**						
	Exercise per day	0.21 (0.34)	0.94 (1.24)	14.191 (62)	<.001	<.001
	Quiz game per day	0.35 (0.41)	1.85 (1.8)	3.253 (62)	<.001	<.001
	Hyodol performance	4.37 (0.57)	4.7 (0.23)	3.867 (62)	.05	.22
	Hyodol satisfaction	2.68 (0.37)	2.8 (0.22)	1.197 (62)	.28	.61

Cluster B users are predominantly women older than 80 years (n=12, 92%). Slightly over half (n=7, 53%) rated their perceived health status as poor, and many also spend a lot of time alone (n=8, 61%). The users in cluster B lead relatively unhealthy lives, as evidenced by the life management survey items. These users are healthier when it comes to the number of diseases. These users have more and longer interactions with SARs and report better physical health. Since they have a high level of interaction with robots throughout the study, we will refer to this group as “SARs as Friends.” When compared to the peers in “SARs as Helpers,” the users in “SARs as Friends” were more likely to be older women who spend more time alone and have a regular meal schedule. However, these are not statistically significant.

As shown in Table 6, users in the “SARs as Friends” group used SARs more frequently per day (t_62_=6.5; *P*=.01) and had a higher retention rate (t_62_=8.2; *P*=.006). In terms of interaction, users in the “SARs as Friends” group were more likely to hold hands with SARs (t_62_=10.6; *P*=.002) and pet them (t_62_=7.3; *P*=.009). For robot-assisted content, users in the “SARs as Friends” group also used SARs for exercise (t_62_=8.6; *P*=.005) and brain training game (t_62_=6.7; *P*=.02). However, SAR performance and satisfaction with Hyodol were found statistically not significant.

#### Content Use

The profiles of clusters C and D users are summarized in Table 7. Cluster C users are welfare recipients, accounting for 59% (n=29). Many of them rated their perceived health status as poor (n=20, 41%), and they spent less time alone. They exhibit balanced lives according to life management surveys, yet with less reliance on SAR content. For the rest of the paper, we will refer to this group as “Short-term Users.”

Cluster D users were mostly older women (n=14, 93%), with an average age of 80.2 (SD 8.4) years. In terms of their perceived health status, fewer healthy users were reported (n=4, 27%), but they have a good social life (n=14, 93%). Users in cluster D are social support recipients (n=7, 47%). In other words, physical difficulties and disabilities translate into poor physical health. To sum up, the users who use SAR contents more and longer are physically unhealthy. For the rest of the paper, we will refer to this group as “Long-term Users.”

In comparison to short-term users, long-term users also demonstrated more frequent use (ie, use day or retention period) of the content feature during the study (t_62_=8.9; *P*=.004) as shown in Table 8. On average per day, long-term users used SARs for exercise (t_62_=14.2; *P*<.001) and brain training features (t_62_=3.3; *P*<.001) more frequently. A higher level of interaction was also observed in terms of handholding (t_62_=16.58; *P*<.001) and petting (t_62_=9.83; *P*=.003).

## Discussion

### Principal Findings

SARs are being seen as a way to help health care professionals improve the quality of care for socially isolated older adults. However, there are challenges in meeting the needs of different types of older adults. This study expands the scope of research in SARs by demonstrating a set of processes through which use data can be used to inform the most effective use for different types of users. Specifically, we adopted time series clustering and profiling to analyze SAR use patterns and highlight use differences among user groups (eg, exercise or fitness and brain training), despite statistically similar demographics, environments, and psychological health.

### Comparison to Prior Work

Several studies conducted in Korea have demonstrated a significant decrease in depression scores among solo-living older individuals who have used the Hyodol robot, suggesting its potential as a supportive companion for solitary older adults [12,14,30]. While these studies have highlighted the potential of SARs in elder care, some raised concerns about a lack of diversity in the participants within their research [31]. This can limit the generalizability of findings and potentially hinder the effectiveness of SARs for a wider range of older adults. Across various fields, data-driven approaches have proven successful in revealing user psychological states [32] and various challenges [33-35].

In recognition of this gap, this study aimed to expand on existing SAR research by explicitly considering the diverse needs of different populations of older people. By incorporating data-driven approaches, we develop a deeper understanding of how SARs can be most effectively tailored to meet the preferences of various user groups and how to identify these users.

### Strengths and Limitations

The findings from this study suggest the importance of differentiating user groups in SAR development. Our finding suggests that tailoring features based on use patterns has the potential to significantly enhance the effectiveness of SARs. Additionally, our experience emphasizes the need for training and support programs for older adults and their caregivers. By providing such resources, we can maximize the benefits that SARs can offer.

We acknowledge the limitations of this study and recommend further research. First, further cluster segmentation based on user patterns can be studied. SAR user patterns can be either cyclical or irregular, and a wide range of these patterns can be further subdivided. Therefore, an increased number of clusters and various classification techniques will facilitate the process of in-depth research on user attitudes and satisfaction depending on use patterns and the effectiveness of SARs.

Second, multifaceted profiling is needed. Due to the small sample size of participants, this study used profiling with a focus on descriptive statistics on demographics and use patterns. Follow-up studies with larger sample sizes can unlock details of use patterns with respect to the effective use of SARs using diverse statistical techniques, including survival analysis. Additionally, understanding outcomes based on different types of SARs and user populations, such as individuals from different cultures, can strengthen our findings.

### Future Directions

These findings have important implications for the development and use of SARs in the future. It is important to consider the needs of different groups of older adults when developing SARs. Effective SARs require tailoring features to specific older adult groups, as use patterns vary dramatically as shown in this study.

Additionally, it is important to provide training and support to older adults and their caregivers on how to use SARs effectively. This will help to ensure that SARs are used in a way that meets the needs of older adults and their caregivers. Several studies conducted in Korea have demonstrated public health social workers who managed the Hyodol care system also highlighted the positive impact of Hyodol in providing companionship, care, and emotional support, particularly in situations where clients felt isolated or lacked regular family interactions [36].

Overall, this study suggests that SARs have the potential to be a valuable tool for meeting the needs of older adults. However, more research is needed to develop SARs that are tailored to the needs of different groups of older adults and to address the practical concerns that have been raised.

### Conclusions

This study categorized users with similar characteristics into clusters based on SAR use patterns. Then, profiling was used to identify and define each cluster’s noteworthy features. Specifically, we used time series SAR use to cluster “Interaction” and “Contents” into 2 use patterns, respectively. We then explored the key characteristics of each use pattern by applying profiling analysis. Despite statistically similar demographics, environments, and psychological health, older adults interact with SARs differently. Finally, based on profiling, we produced various insights into the use of SARs. This study extends our understanding of the factors associated with the long-term use of SARs for geriatric care and makes methodological contributions.

## Appendix

Multimedia Appendix 1 A TED Talk for a further exploration of Hyodol SAR and its applications.

Multimedia Appendix 2 Investigator-developed questionnaires and measurement instruments used in this study.

## Abbreviations

GAK: global alignment kernel
SAR: socially assistive robot

## Data Availability

The datasets generated during or analyzed during this study are not publicly available due to privacy concerns for the participants.
