# Supplementary material for: Investigating Older Adults' Use of a Socially Assistive Robot via Time Series Clustering and User Profiling: Descriptive Analysis Study
Source: JMIR Form Res. 2024 Sep 19;8:e41093. doi: 10.2196/41093 (PMC11450348; doi:10.2196/41093)
Supplement: Multimedia Appendix 2 [file formative_v8i1e41093_app2.docx]

**Multimedia Appendix 1: Investigator-developed questionnaires and measurement instruments**

**Physical Health**

| How would you rate your own health status? | ① Not healthy ② Average ③ Healthy |
| --- | --- |
| How much time do you spend alone in a day? | ① Short period alone ② Average ③ Long period alone |
| Please tell us about any illnesses you currently have. (Multiple responses possible) | ① None ② Hypertension ③ Hyperlipidemia ④ Diabetes ⑤ Arthritis/Osteoporosis/Back Disc ⑥ Stroke/Cerebral Hemorrhage ⑦ Heart Disease ⑧ Respiratory Disease ⑨ Digestive Disease ⑩ Urinary Disease ⑪ Cancer ⑫ Eye Disease ⑬ Other ( ) |

**Lifestyle Management**

| Do you wake up/go to bed at the same time every day? | ① Yes, regularly every day  ② Sometimes  ③ Not regularly |
| --- | --- |
| Do you ventilate your room every day? | ① Yes, regularly every day  ② Occasionally, when needed  ③ Rarely |
| Do you take your medication at the exact same time every day? | ① Yes, at the exact time every day  ② Sometimes I forget to take it  ③ I often forget to take it |
| Do you have three regular meals a day? | ① Yes, three regular meals every day  ② Sometimes I miss a mealtime  ③ I often miss mealtimes |
| How often do you go for a walk? | ① Regularly every day  ② Occasionally, when needed  ③ Rarely |
| How often do you do exercises? (Including indoor exercises) | ① Regularly every day  ② Occasionally, when needed  ③ Rarely |
| How often do you have positive thoughts? | ① Positive every day  ② Sometimes I have negative thoughts  ③ Rarely have positive thoughts |
| How often do you feel like contacting others (via phone, meetings, etc.)? | ① Want to contact others every day  ② Sometimes want to contact others  ③ Do not want to contact others |

**Mental Health – Depression**

| Are you generally satisfied with your current life? | ① Yes ② No |
| --- | --- |
| Have you noticed a significant decrease in your activity level or motivation recently? | ① Yes ② No |
| Do you feel that you are living your life in vain? | ① Yes ② No |
| Do you often find life boring? | ① Yes ② No |
| Is your mood usually refreshing? | ① Yes ② No |
| Do you feel anxious about bad things happening to you? | ① Yes ② No |
| Are you generally happy? | ① Yes ② No |
| Do you frequently feel despair? | ① Yes ② No |
| Do you dislike going outside and prefer staying at home? | ① Yes ② No |
| Do you feel that your memory is worse compared to other seniors of your age? | ① Yes ② No |
| Do you find joy in being alive at the moment? | ① Yes ② No |
| Do you feel like you are a useless person now? | ① Yes ② No |
| Do you consider yourself to be energetic? | ① Yes ② No |
| Do you feel that your current situation is hopeless? | ① Yes ② No |
| Do you feel that you are worse off than others? | ① Yes ② No |

**Performance evaluation**

| Having Hyodol, I don't feel lonely or isolated. | ① Strongly disagree ② Disagree ③ Neutral  ④ Agree ⑤ Strongly agree |
| --- | --- |
| With Hyodol, I'm never bored. | ① Strongly disagree ② Disagree ③ Neutral  ④ Agree ⑤ Strongly agree |
| Hyodol helps me remember to take my medication and eat meals on time. | ① Strongly disagree ② Disagree ③ Neutral  ④ Agree ⑤ Strongly agree |
| Thanks to Hyodol, I can easily do exercises and dementia prevention programs at home. | ① Strongly disagree ② Disagree ③ Neutral  ④ Agree ⑤ Strongly agree |
| Hyodol contributes to my overall healthy lifestyle. | ① Strongly disagree ② Disagree ③ Neutral  ④ Agree ⑤ Strongly agree |
| Hyodol brings vitality to my life. | ① Strongly disagree ② Disagree ③ Neutral  ④ Agree ⑤ Strongly agree |
| I feel more secure with Hyodol than without it. | ① Strongly disagree ② Disagree ③ Neutral  ④ Agree ⑤ Strongly agree |
| Overall, my life is more satisfying with Hyodol compared to without it. | ① Strongly disagree ② Disagree ③ Neutral  ④ Agree ⑤ Strongly agree |
| Hyodol feels friendly to me. | ① Strongly disagree ② Disagree ③ Neutral  ④ Agree ⑤ Strongly agree |
| Hyodol helps me stay better connected with my family and caregivers. | ① Strongly disagree ② Disagree ③ Neutral  ④ Agree ⑤ Strongly agree |

**Satisfaction**

| Hyodol is easy to use. | ① Strongly disagree ② Disagree ③ Neutral  ④ Agree ⑤ Strongly agree |
| --- | --- |
| I like the appearance/size of Hyodol. | ① Strongly disagree ② Disagree ③ Neutral  ④ Agree ⑤ Strongly agree |
| I like what Hyodol says. | ① Strongly disagree ② Disagree ③ Neutral  ④ Agree ⑤ Strongly agree |
| I like the texture of Hyodol. | ① Strongly disagree ② Disagree ③ Neutral  ④ Agree ⑤ Strongly agree |
